# Primary Thyroid Dysfunction Is Prevalent in Hidradenitis Suppurativa and Marked by a Signature of Hypothyroid Graves’ Disease: A Case–Control Study

**DOI:** 10.3390/jcm12237490

**Published:** 2023-12-04

**Authors:** Nessr Abu Rached, Johannes W. Dietrich, Lennart Ocker, Daniel R. Quast, Christina Scheel, Thilo Gambichler, Falk G. Bechara

**Affiliations:** 1 International Centre for Hidradenitis Suppurativa/Acne Inversa (ICH), Department of Dermatology, Venereology and Allergology, Ruhr-University Bochum, 44791 Bochum, Germany; lennart.ocker@kklbo.de (L.O.); christina.scheel@kklbo.de (C.S.); falk.bechara@kklbo.de (F.G.B.); 2 Skin Cancer Center, Department of Dermatology, Venereology and Allergology, Ruhr-University Bochum, 44791 Bochum, Germany; t.gambichler@hospitalverbund.de; 3 Diabetes, Endocrinology and Metabolism Section, Department of Internal Medicine I, St. Josef Hospital, Ruhr University Bochum, NRW, Gudrunstr. 56, 44791 Bochum, Germany; johannes.dietrich@ruhr-uni-bochum.de (J.W.D.);; 4 Diabetes Centre Bochum-Hattingen, St. Elisabeth-Hospital Blankenstein, Im Vogelsang 5-11, 45527 Hattingen, Germany; 5 Centre for Rare Endocrine Diseases, Ruhr Centre for Rare Diseases (CeSER), Ruhr University Bochum and Witten/Herdecke University, Alexandrinenstr. 5, 44791 Bochum, Germany; 6 Centre for Diabetes Technology, Catholic Hospitals Bochum, Gudrunstr. 56, 44791 Bochum, Germany; 7 Department of Dermatology and Phlebology, Christian Hospital Unna, 59423 Unna, Germany

**Keywords:** hidradenitis suppurativa, HS, acne inversa, hormones, obesity, thyroid function, autoimmunity, thyroxine, triiodothyronine, SPINA-GT, SPINA-GD

## Abstract

Hidradenitis suppurativa (HS) is a chronic skin disease that can have an association with endocrine disorders. There is conflicting information in the literature regarding the role of the thyroid gland in HS. This study aimed to close this knowledge gap and investigate how thyroid disease is involved in patients with HS. We carried out a case–control study with a total of 160 patients, of whom 108 were patients with HS and 52 were controls matched for age and sex. Parametric and non-parametric methods were used to analyze the results. We calculated structural parameters of thyroid homeostasis to detect subclinical thyroid disease, non-thyroid disease syndrome and other forms. The severity of HS was not associated with thyroid hormone levels and antibodies (*p* > 0.05). HS patients with or without hypothyroidism had decreased FT4 levels and a decreased thyroid secretory capacity (SPINA-GT). Titers of TSH receptor autoantibodies (TRAb) were significantly higher in smoking HS patients compared to non-smokers (median: 1.18 vs. 1.08; *p* = 0.042). The rate of subclinical hypothyroidism was significantly higher in HS patients; thus, subclinical hypothyroidism is an important comorbidity of HS (*p* < 0.05). Further studies are needed to investigate whether the chronic inflammation of HS is a cause of increased rates of (subclinical) hypothyroidism.

## 1. Introduction

Hidradenitis suppurativa (HS) is a chronic skin disease characterized by recurrent inflamed abscesses, fistulae and scarring of the inverse skin areas [1,2,3]. Typically, the axilla, genital, submammary, inguinal, perianal and gluteal regions are affected in HS. HS is thought to have a multifactorial pathogenesis consisting of a combination of genetic, immunological, environmental and hormonal factors [4,5,6,7,8]. Treatment for HS includes antibiotics, biologics, surgery and local therapies [9,10]. Comorbidities include metabolic syndrome, diabetes mellitus, inflammatory bowel disease, thyroid dysfunction, acne vulgaris and rheumatological and psychiatric comorbidities [11,12,13,14,15]. However, the relationship between thyroid disease (hyperthyroidism or hypothyroidism) and HS is currently controversial. Hypothyroidism is more common in HS than in the general population and healthy controls, according to recent meta-analyses [16,17]. There is limited evidence that thyroid hormones may be involved in the inflammatory response [18]. Some studies have shown that thyroid hormones such as triiodothyronine (T3) and thyroxine (T4) can influence immune system function by inhibiting or enhancing inflammation [19,20]. HS is a disease characterized by chronic inflammation. For example, the level of haptoglobin, an acute-phase protein, correlates with the severity of HS [21]. It is assumed that autoimmunity plays an important role in HS [22,23].

The aim of our study was to determine the prevalence of primary or secondary thyroid dysfunction in HS and compare it to a matched control group. In addition, this study aimed to investigate whether there is evidence that autoimmune thyroid disease is more common in HS. To our knowledge, functional structural parameters have not yet been used to detect subclinical thyroid dysfunction in HS patients. We calculated the thyroid’s secretory capacity (SPINA-GT), total step-up deiodinase activity (SPINA-GD), Jostel’s TSH index (TSHI) and the thyrotroph thyroid hormone sensitivity index (TTSI) according to established methods.

## 2. Materials and Methods

### 2.1. Study Design

This cross-sectional, case–control study was designed to investigate the prevalence of primary or secondary thyroid disease in patients with HS and the possible signatures of thyroid dysfunction in this setting. Investigating and collecting the data started in February 2022 and ended in February 2023. For this study, the Diabetes, Endocrinology and Metabolism Section Bochum and the International Centre for Hidradenitis suppurativa/Acne inversa (ICH) Bochum collaborated. A study flowchart can be found in Appendix A.

### 2.2. Patients and Data Collection

Our study involved 108 patients with HS, and 52 age- and sex-matched control subjects participated without HS. The diagnosis of HS was given by two experienced dermatologists using the Dessau criteria [24]. All patients underwent blood sampling with laboratory concentrations for TSH, FT3, FT4 and autoantibodies, including anti-TPO (TPO-Ab), TSH receptor autoantibodies (TRAb) and anti-thyroglobulin (Tg-AB). Data on comorbidities, medications and personal characteristics were collected during the visit. In addition, HS patients had their lesions counted at the visit to determine their Severity Assessment of Hidradenitis Suppurativa (SAHS) score [25], modified Hidradenitis Suppurativa Score (mHSS) [26] and Hurley stage [27]. Quality of life was assessed using the Dermatology Life Quality Index (DLQI) [28]. All patients with confirmed HS disease according to the established Dessau criteria were included in the HS group [24]. For both groups, the minimum age required to participate was 18 years. People without HS or other inflammatory diseases (psoriasis vulgaris, Crohn’s disease, etc.) were included in the control group. Otherwise, there were no further exclusion criteria for the control group in order to obtain a representative collective. Informed consent was obtained from all subjects involved in the study. This study was conducted in accordance with the Declaration of Helsinki and approved by the Ethics Committee of Ruhr-Universität Bochum (protocol code: 5076–14; date of approval: 30 June 2014).

Our control group was a matched group with a high BMI comparable to that of HS patients (BMI > 30). For example, according to Mahdavi et al., the prevalence of hypothyroidism in obese people is 11.6% (p0). According to Sherman et al. [29], there is an odds ratio of 2.91 for hypothyroidism and 2.21 for hyperthyroidism in HS, so the expected odds ratio for thyroid disease will be significantly higher than 2.91. The sample size was calculated using established methods [30,31]. To calculate the sample size, a type I error rate (*α*) of 0.05, a power (1-*β*) of 0.8, a case–control ratio (k) of 0.5, a correlation coefficient (r) of 0.3, an odds ratio (OR) of 2.91 and p0 = 0.116 were assumed. Based on the calculation, at least 92 cases and 46 matched controls were required.

### 2.3. Laborarory Investigation

Serum concentrations of TSH, fT4 and fT3 as well as titers for thyroid tissue autoantibodies (TPOAb, TgAb and TRAb) were determined with a fully automated chemiluminescence-based system (Elecsys TSH, Elecsys FT4 II, Elecsys FT3 III, Elecsys Anti-Tg, Elecsys Anti-TPO and Elecsys Anti-TSHR for the cobas 801 analyzer, Roche Diagnostics, Mannheim, BW, Germany). Determinations were performed in accordance with the guidelines of the German Medical Association (Bundesärztekammer) for quality control of the laboratory investigations. The intra-assay and inter-assay CVs for these analyses vary with concentrations but are <20% for the range of measurement.

Comorbidities were screened for pre-existing hypo- or hyperthryroidism. Primary hyperthyroidism was defined as a low TSH level (<0.27 µU/mL). The patients were also asked about their general symptoms of hyperthyroidism (e.g., weight loss, restlessness, tachycardia) or hypothyroidism (e.g., weight gain, fatigue). Primary hypothyroidism was defined as an elevated TSH level (>4.2 µU/mL).

### 2.4. Calculation of Structural Parameters of Thyroid Homeostasis

Structural parameters of thyroid homeostasis were calculated using an established methodology [32], as recently recommended for thyroid trial design [33,34]. The use of structural parameters provides a more integrated and systemic view, particularly on the physiology of feedback control between the pituitary and thyroid glands [32], and helps to identify central and peripheral mechanisms underlying observed variations in thyroid hormones. We therefore calculated the thyroid’s secretory capacity (SPINA-GT), total step-up deiodinase activity (SPINA-GD), Jostel’s *TSH* index (*TSHI*) and the thyrotroph thyroid hormone sensitivity index (*TTSI*).

The SPINA-GT was determined with

$${\hat{G}}_{T}=\frac{\beta_{T}\left(D_{T}+[TSH]\right)\left(1+K_{41}\left[TBG\right]+K_{42}[TTR]\right)[{FT4}_{}]}{\alpha_{T}[TSH]}$$

from concentrations of *TSH*, FT4 and constant parameters for plasma protein binding, distribution and elimination, as previously described [32].

Likewise, the SPINA-GD was obtained with

$${\hat{G}}_{D}=\frac{\beta_{31}\left(K_{M1}+[F{T4}_{}]\right)\left(1+K_{30}[TBG]\right)[F{T3}_{}]}{\alpha_{31}[F{T4}_{}]}$$

from concentrations of FT3, FT4 and constants for the kinetics of hormones [32].

Parameters for the central function of the feedback loop (the so-called set point) included Jostel’s *TSH* index [35],

$$TSHI=\ln\left(\left[TSH\right]\right)+\beta [F{T4}_{}]$$

and the *TTSI*,

$$TTSI=\frac{100\left[TSH\right][FT4]}{l_{U}}$$

Numerical values for the parameters are described in the literature [32]. The SPINA-GT was not calculated for subjects on replacement therapy with L-thyroxine.

### 2.5. Statistical Analysis

All variables were tested for normal distribution. Parametric and non-parametric tests were used to identify differences between the groups. We also performed a subgroup analysis of euthyroid HS patients compared with non-HS controls to investigate whether the association varied. The statistical analyses were performed using the R software (R Core Team, version 4.3.1, Vienna, Austria, 2022) and IBM SPSS Statistics (version 29.0.0.0, New York, United States of America, 2022). A significance level of *p* < 0.05 was considered statistically significant.

## 3. Results

### 3.1. Personal and Clinical Characteristics of HS Patients

A total of 108 HS patients were included in this study. Table 1 provides an overview of the personal and clinical characteristics of the HS study population. The mean BMI indicates a variable distribution of body mass within the cohort, with a mean of 31.4 kg/m² and a standard deviation (SD) of ±6.5. Our findings include a nearly even distribution of males and females (53.7% vs. 46.3%), a mean ± SD age of 42 ± 13 years and a median disease duration of 13.5 years (interquartile range, IQR, 6–24.3). The median mHSS score for all patients is 40.5 (IQR 21–71.3), indicating moderate disease severity on average. Approximately 30% of patients (n = 33) have a positive family history of HS, while 70% have a negative family history (n = 75). Hypothyroidism is present in 17.6% in HS, with variations between males (10.3%) and females (26%). At the time of the visit, all HS patients with hypothyroidism were receiving replacement levothyroxine (L-T4). In the HS study group, 64.8% are current smokers, 1.9% are former smokers and 33.3% are non-smokers, indicating a wide range of smoking behavior.

### 3.2. Comparison of HS Patients with Control Group

Table 1 and Table 2 provide a detailed comparison of the personal and thyroid characteristics between the two distinct groups: HS patients (n = 108) and a matched control group (n = 52). Firstly, the mean age for the HS patients was 42 ± 13 years, while the control group had a slightly higher mean age of 43.2 ± 9.8 years, with a non-significant *p*-value of 0.65, indicating no substantial age difference between the groups. In terms of gender distribution, 53.7% of the HS patients were male, while 50% of the control group was male, so the control group was suitable for comparison. The HS patients had a significantly higher mean BMI of 31.4 ± 6.5 kg/m² compared to the control mean of 27 ± 4.9 kg/m^2^ (*p* < 0.001). There was a striking difference in smoking status, with a clear majority of HS patients (64.8%) being current smokers, whereas only a small proportion (9.6%) of the controls smoke. For hypothyroidism, the HS patients had a higher prevalence (17.6%) than the controls (7.7%). The thyroid-related markers include TSH, fT3, fT4, TRAb, TPO-Ab and Tg-Ab levels. Among these, the fT4 levels showed a statistically significant difference, with the HS patients having lower median fT4 levels than the control group (1.24 vs. 1.34; *p* = 0.016). the TRAb levels were significantly higher in the HS patients compared to the controls (median: 1.08 vs. 0.8; *p* < 0.001). Furthermore, smokers and ex-smokers had significantly higher TRAb levels than non-smoking HS patients (median: 1.18 vs. 1.08; *p* = 0.042). In addition, the TgAb levels were significantly higher in the HS patients than the controls (median: 15.4 vs. 14.2; *p* = 0.026). However, at time of visit, no HS patient had Graves’ disease or another form of hyperthyroidism. The TPO-Ab levels did not differ between the two groups (*p* = 0.05). There were no differences between smokers and non-smokers in terms of their TPO-Ab and Tg-Ab thyroid antibodies or thyroid levels (*p* > 0.05).

### 3.3. Comparison of Characteristics from HS Patients with and without Hypothyroidism

Table 3 presents a comprehensive comparison of various characteristics between HS patients who had hypothyroidism and those without hypothyroidism. The HS patients with hypothyroidism had a longer median HS disease duration of 23 years (ICR 12–30.5) compared to patients without hypothyroidism, who had a median disease duration of 12 years (ICR 2–24). This difference was statistically significant (*p* = 0.018). Compared to patients without hypothyroidism (68.4% vs. 41.6%; *p* = 0.033), a higher percentage of patients with hypothyroidism were female. The two groups showed no significant difference in terms of their SAHS score, mHSS score, Hurley stage, DLQI and BMI (*p* > 0.05). Patients with hypothyroidism had significantly lower median TSH, FT3 and FT4 concentrations compared to those without hypothyroidism (*p* = 0.001, <0.001, and <0.001, respectively). However, there were no statistically significant differences between the two groups in the titers of TRAb, TPO-Ab and Tg-AB.

### 3.4. Comparison of Euthyroid HS Patients with Healthy Controls

**Figure 1:**
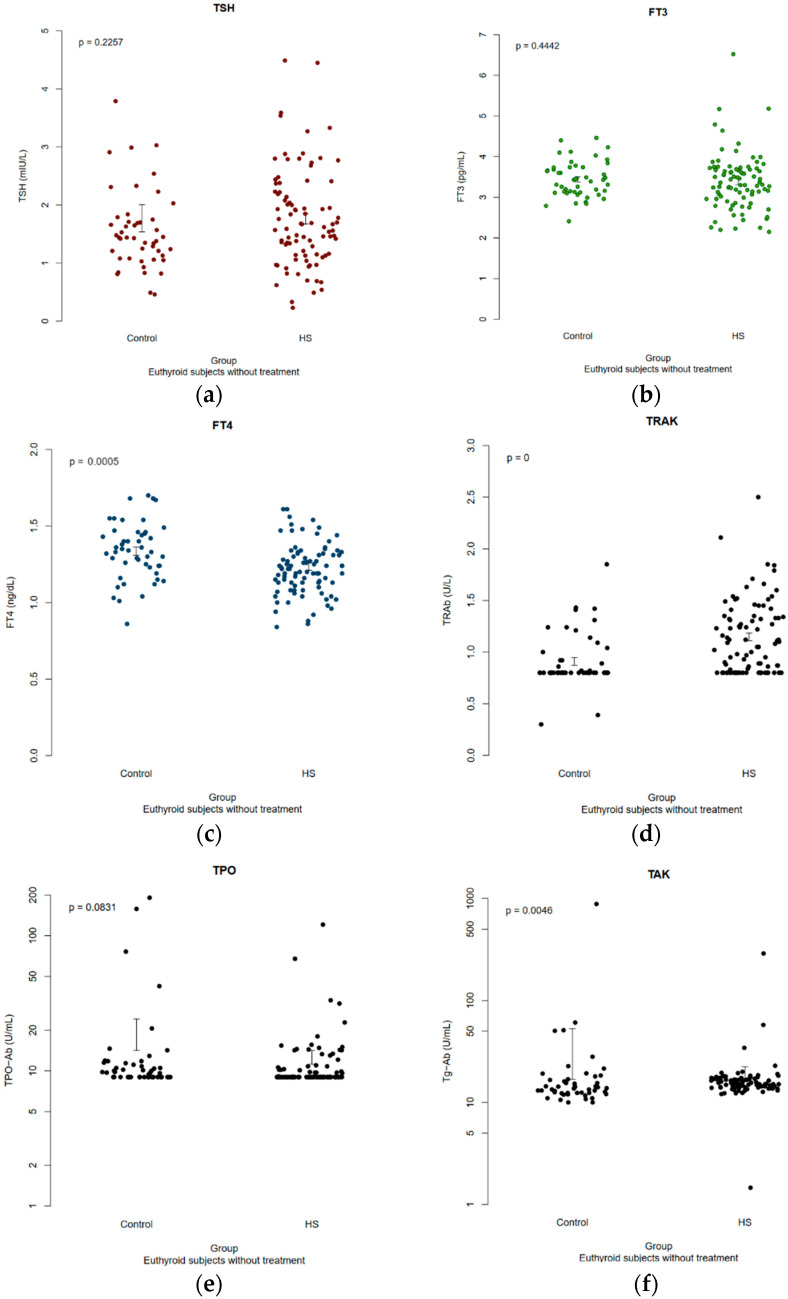
Scatter plot with superimposed symbols for the standard error of the mean (SEM) and the effect size of various thyroid parameters in euthyroid patients without treatment; left are controls and right are HS patients: (**a**) TSH, thyroid-stimulating hormone; (**b**) Ft3, free triiodothyronine; (**c**) FT4, free thyroxine; (**d**) TRAb, thyrotropin receptor autoantibody; (**e**) TPO-Ab, thyroperoxidase antibody; (**f**) Tg-Ab, thyroglobulin antibody.

In a further subanalysis, we included only euthyroid HS patients and controls to minimize the influence of iatrogenic thyroxine depletion. In a comparative analysis between euthyroid HS patients (n = 89) and euthyroid healthy controls (n = 48), their thyroid levels and antibodies were analyzed (Table 4 and Figure 1). The euthyroid HS patients also had significantly higher mean BMIs (30.9 kg/m^2^) than the healthy euthyroid controls (30.9 vs. 26.8 kg/m^2^; *p* < 0.001). Notably, the euthyroid HS patients had significantly lower FT4 concentrations than the euthyroid healthy controls (*p* < 0.001). There was no significant difference in the levels of either FT3 or TSH between the two groups (*p* > 0.05). In addition, the euthyroid HS patients had significantly higher TRAb and Tg-Ab titers than the healthy euthyroid controls (*p* < 0.001 and *p* = 0.005, respectively), but there was no difference in their TPO-Ab titers.

### 3.5. Results of Structural Parameters of Thyroid Homeostasis

**Figure 2:**
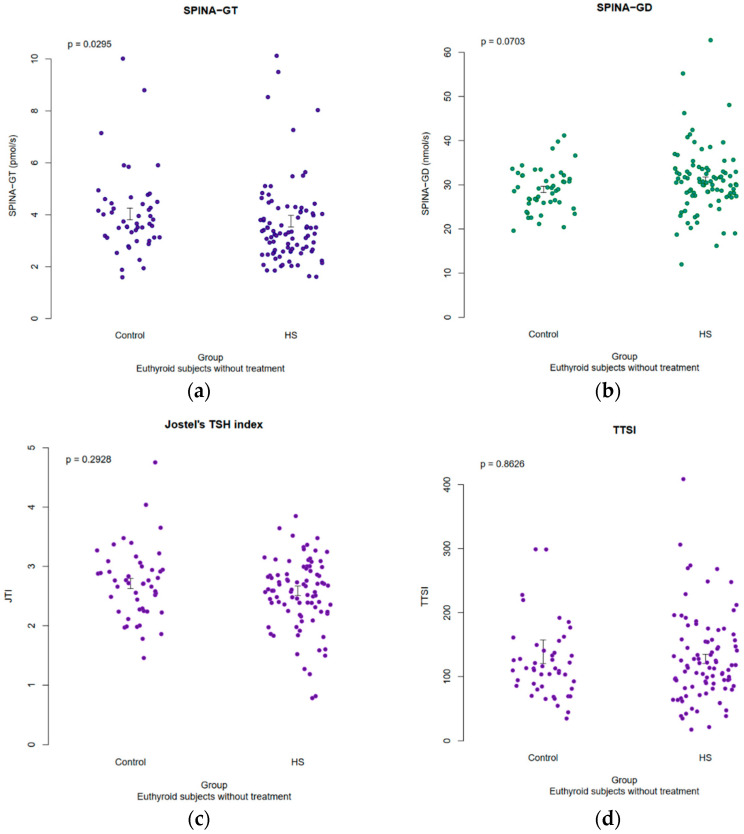
Scatter plot with superimposed symbols for the standard error of the mean (SEM), mean and the effect size of the calculated structural thyroid parameters: (**a**) SPINA-GT, secretory capacity of the thyroid gland; (**b**) SPINA-GD, sum activity of peripheral deiodinase; (**c**) TSHI, Jostel’s TSH index; (**d**) TTSI, thyrotroph thyroid hormone resistance index.

The results of the thyroid homeostasis are visible in Figure 2. Compared to the euthyroid healthy controls, the HS patients had a significantly lower SPINA-GT, which means that the secretory capacity of their thyroid gland is reduced (subjects not on replacement therapy with levothyroxine only, *p* < 0.05). There was no difference between the euthyroid HS patients and healthy controls in terms of their SPINA-GD, Jostel’s TSH index and TTSI (*p* > 0.05).

## 4. Discussion

HS is an inflammatory skin disease, with recent evidence for systemic inflammation affecting multiple organs. In particular, the thyroid gland, a central organ in endocrinology involved in various metabolic pathways, is commonly affected by inflammatory and autoimmune diseases. Hypothyroidism is more common in HS patients than in the normal population, as confirmed through several meta-analyses [16,17]. Our results also showed that hypothyroidism was more common in HS patients than in the control group (17.6% vs. 7.7%). Recent analyses from both Germany and Europe suggest a population prevalence for primary hypothyroidism of about 3% [36,37]. In the SHIP-TREND study, this also applied to the age group between 40 and 49 years, which corresponds to the mean of our cohorts. Therefore, the difference is even more pronounced on the larger level of nations and continents. Hyperthyroidism was not found in any of the HS patients. Other studies have also shown a limited importance of hyperthyroidism in HS [38]. Previous studies have suggested that smoking is a factor in hyperthyroidism. However, our study did not confirm this association. To our knowledge, no meta-analysis has, until now, shown an increased incidence of Graves’ disease or other hyperthyroidism.

The role of thyroid antibodies in HS also remains controversial. A study by González-López et al. showed that there was no statistically significant difference between HS patients and controls in the prevalence of thyroid antibodies [39]. González-López et al. concluded that conventional autoimmune mechanisms may not play a role in the development of HS [39]. To detect a possible association between autoimmune thyroiditis and HS, we also measured thyroid antibodies TRAb, TPO-Ab and Tg-AB. Surprisingly, although hypothyroidism is more common in HS patients, an elevation of TPO-Ab could not be shown. Hence, we concluded that the underlying pathogenesis of hypothyroidism in HS patients may be different from that in Hashimoto’s thyroiditis. However, patients with HS have higher titers of TRAb and lower titers of Tg-Ab in comparison to healthy control patients. Previous studies have shown a correlation between TRAb antibody levels and smoking status [40]. There was also a significant association between smokers (including ex-smokers) and non-smokers in our cohort. Smokers and ex-smokers had significantly higher TRAb levels than non-smoking HS patients (*p* = 0.042). The relationship between TRAb levels and hypothyroidism is unclear. In Graves’ disease, both inhibitory and excitatory TRAb antibodies have been observed, so that the importance of TRAb antibodies cannot be conclusively assessed [41]. Further investigation will require assays that can differentiate between TRAb subtypes [42,43]. Alternative explanations include the direct effects of cytokines on thyroid function; e.g., interferon alpha and IL-2 were demonstrated to elicit hypothyroidism. Of note, the interaction between thyroid function and the immune response is complex and bidirectional [44,45,46,47,48].

For the detection of subclinical thyroid disease and to assess the relative contributions of the thyroid, the pituitary gland and peripheral endocrine organs to the phenotypical variation in free T4 concentration, we calculated structural parameters of thyroid homeostasis. We observed that the thyroid’s secretory capacity (SPINA-GT) was significantly lower in euthyroid HS patients compared to euthyroid controls, whereas calculated biomarkers for deiodinase activity, pituitary function and the central set point of the homeostatic system were identical. This suggests that the predominant etiology of hypothyroidism in HS patients is of a primary nature, i.e., functional failure of the thyroid gland.

Chronic inflammation could be a possible cause of the tendency for hypothyroidism and decreased thyroid secretion [49]. Inflammatory markers in the blood correlate with the clinical HS severity [21,50,51,52]. Chronic inflammation could lead to inflammatory damage of the thyroid gland, with subsequent hypothyroidism. Our analysis also showed a significant association between the presence of hypothyroidism and a long duration of HS, so chronic inflammation could be a cause of hypothyroidism. The chronic release of cytokines triggers inflammatory cascades that can lead to changes in thyroid tissue, altering its function. Likewise, an acute upregulation of proinflammatory cytokines is assumed to facilitate autoimmune thyroiditis in viral infections, including COVID-19 and Epstein–Barr virus infection [53,54,55,56]. Similar effects on endocrine organs, particularly the thyroid gland, are known from the use of PD1 or CTLA4 inhibitors in the treatment of skin cancer [57,58,59,60]. PD1 and CTLA-4 inhibitors increase the activity of the immune system, which can lead to thyroid dysfunction [61,62,63,64,65,66]. Dysregulation of the immune response is also a suspected cause of HS [7,67].

Summarizing the results of the phenotypical classification of thyroid function and autoantibody determination, we assume that the observed high prevalence of hypothyroidism in HS patients may be caused by hypothyroid Graves’ disease (type 3C autoimmune thyroiditis [68]) rather than by Hashimoto’s or Ord’s disease. Another explanation may be the induction of secondary Graves’ disease in the setting of thyroiditis due to non-specific inflammation [69], as recently described for COVID thyroiditis [70]. HS patients with hypothyroidism are significantly more likely to be female (female proportion with and without hypothyroidism: 68.4% vs. 42.6%; *p* = 0.033). However, the higher prevalence and incidence of thyroid dysfunction in women may explain the difference [37].

In addition, HS patients showed significantly increased BMIs compared with healthy subjects, which we expected. This well-known observation may result from hyperinsulinism, allostatic load or, as suggested by our study, hypothyroidism. It is currently being debated if obesity is a cause or consequence of hypothyroidism. Several studies have found simultaneous elevations of TSH and T3 concentrations and SPINA-GD in obesity, pointing to common mechanisms (e.g., type 2 allostatic load) for both overweight and elevated thyrotropin concentration [71]. Further studies are needed to investigate the effects of anti-inflammatory treatment on HS and thyroid function.

One limitation of this study is that the thyroid levels were only measured at one time. Further studies should prospectively analyse thyroid levels over multiple time points. It is also interesting to correlate the level of inflammation (e.g., haptoglobin or CRP) with the presence of thyroid dysfunction. An interesting endpoint for further studies is the effect of anti-inflammatory HS therapies (e.g., adalimumab and secukinumab) on thyroid dysfunction.

## 5. Conclusions

HS patients with and without hypothyroidism have decreased FT4 levels compared to healthy controls. Hypothyroidism is a common comorbidity of HS caused by a reduced thyroid secretory capacity (SPINA-GT). The pathomechanism of reduced thyroid secretion remains to be investigated, in particular the influence of chronic inflammation and meta-inflammation on the thyroid. Thyroid hormone concentrations or antibodies are not related to the severity of HS, but hypothyroidism is related to the duration of HS. TRAb levels are significantly higher in smokers with HS. Hypothyroidism is an important comorbidity of HS, especially in female patients.

## Figures and Tables

**Table 1:** Personal and clinical characteristics of HS patients (n = 108) and control group (n = 52).

Parameter		HS Patient Value (s)	Control Group Value (s)
sex, n (%)	female male	50 (46.3) 58 (53.7)	26 (50) 26 (50)
age, mean (±SD), y		42 (±13)	43.2 (±9.8)
age of onset, median (IQR), y		21 (17–31)	-
disease duration, median (IQR), y		13.5 (6–24.3)	-
BMI, mean (±SD), kg/m^2^		31.4 (±6.5)	27 (±4.9)
family history of HS, n (%)	positive negative	33 (30.6) 75 (69.4)	-
smoker, n (%)	current smoker ex-smoker non-smoker	70 (64.8) 2 (1.9) 36 (33.3)	5 (9.6) 0 (0) 47 (90.4)
hypothyroidism, n (%)	total	19 (17.6)	4 (7.7)
	male female	6 (10.3) 13 (26)	0 (0) 4 (15.4)
prevalence of hypothyroidism to Hurley classification, n (%)	Hurley I Hurley II Hurley III	1 (12.5) 11 (22) 7 (14)	-
mHSS, median (IQR)		40.5 (21–71.3)	-
SAHS, median (IQR)		8 (5–9)	-

n, absolute number of patients; SD, standard deviation; y, years; IQR, interquartile range; BMI, body mass index; HS, hidradenitis suppurativa; mHSS, modified Hidradenitis Suppurativa Score; SAHS, Severity Assessment of Hidradenitis Suppurativa.

**Table 2:** Comparison of the personal and thyroid characteristics of HS patients (n = 108) with a control group (n = 52).

Parameter	HS Patients (n = 108)	Control Group (n = 52)	*p* Value(s)
TSH, median (IQR)	1.49 (0.99–2.15)	1.44 (1.12–1.89)	0.95
FT3, median (IQR)	3.25 (2.8–3.66)	3.41 (3.12–3.71)	0.11
FT4, median (IQR)	1.24 (1.13–1.37)	1.34 (1.24–1.46)	0.016 *
TRAb, median (IQR)	1.08 (0.8–1.35)	0.8 (0.8–0.92)	<0.001 *
TPO-Ab, median (IQR)	9 (9–10.7)	9.8 (9–11.4)	0.05
Tg-Ab, median (IQR)	15.4 (14.05–17)	14.2 (12.4–17.8)	0.026 *

n, absolute number of patients; IQR, interquartile range; HS, hidradenitis suppurativa; TSH, thyroid-stimulating hormone; FT3, free triiodothyronine; FT4, free thyroxine; TRAb, thyrotropin receptor autoantibody; TPO-Ab, thyroperoxidase antibody; Tg-Ab, thyroglobulin antibody; * significant result.

**Table 3:** Comparison of characteristics from HS patients (n = 108) with and without hypothyroidism.

Parameter	HS Patients with Hypothyroidism (n = 19)	HS Patients without Hypothyroidism (n = 89)	*p* Value(s)
Disease duration of HS, median (ICR), y	23 (12–30.5)	12 (2–24)	0.018 *
Initial manifestation of HS, median (ICR), y	20 (18–28)	21 (16–31)	0.6
BMI, mean (± SD), kg/m^2^	30.5 (26.3–36.3)	30.9 (26.8–34.2)	0.7
Female, n (%)	13 (68.4)	37 (41.6)	0.033 *
mHSS, median (IQR)	47 (19–70)	40 (21–74)	0.9
SAHS, median (IQR)	8 (5–11)	8 (5–9)	0.26
DLQI, median (IQR)	15 (9.5–24.5)	13 (7–19)	0.086
Hurley III, n (%)	7 (36.8)	43 (48.3)	0.5
TSH, median (IQR)	0.67 (0.36–1.43)	1.62 (1.15–2.22)	0.001 *
fT3, median (IQR)	2.66 (2.54–3)	3.34 (2.98–3.7)	<0.001 *
fT4, median (IQR)	1.57 (1.32–1.76)	1.22 (1.13–1.32)	<0.001 *
TRAb, median (IQR)	0.97 (0.8–1.34)	1.1 (0.8–1.34)	0.7
TPO-Ab, median (IQR)	9 (9–10.25)	9 (9–10.65)	0.5
Tg-Ab, median (IQR)	14.8 (13.85–16.5)	15.5 (14.1–17.02)	0.5

n, absolute number of patients; SD, standard deviation; y, years; IQR, interquartile range; BMI, body mass index; HS, hidradenitis suppurativa; TSH, thyroid-stimulating hormone; FT3, free triiodothyronine; FT4, free thyroxine; TRAb, thyrotropin receptor autoantibody; TPO-Ab, thyroperoxidase antibody; Tg-Ab, thyroglobulin antibody; mHSS, modified Hidradenitis Suppurativa Score; SAHS, Severity Assessment of Hidradenitis Suppurativa; DLQI, Dermatology Life Quality Index; * significant result.

**Table 4:** Comparison of euthyroid HS patients (n = 189) and euthyroid healthy controls (n = 48).

Parameter	Euthyroid HS Patients (n = 89)	Euthyroid Healthy Controls (n = 48)	*p* Value(s)
Age, mean (±SD), y	40.6 (±13.1)	42.8 (±10.1)	0.24
BMI, mean (±SD), kg/m^2^	30.9 (26.8–34.2)	26.8 (24.4–29.4)	<0.001 *
Female, n (%)	37 (41.6)	22 (45.8)	0.6
TSH, median (IQR)	1.62 (1.15–2.22)	1.44 (1.12–1.76)	0.23
FT3, median (IQR)	3.34 (2.98–3.7)	3.43 (3.13–3.73)	0.45
FT4, median (IQR)	1.22 (1.13–1.32)	1.34 (1.24–1.45)	<0.001 *
TRAb, median (IQR)	1.1 (0.8–1.34)	0.8 (0.8–0.94)	<0.001 *
TPO-Ab, median (IQR)	9 (9–10.65)	9.75 (9–11.43)	0.11
Tg-Ab, median (IQR)	15.5 (14.1–17.02)	13.75 (12.4–16.7)	0.005 *

n, absolute number of patients; SD, standard deviation; y, years; IQR, interquartile range; BMI, body mass index; HS, hidradenitis suppurativa; TSH, thyroid-stimulating hormone; FT3, free triiodothyronine; FT4, free thyroxine; TRAb, thyrotropin receptor autoantibody; TPO-Ab, thyroperoxidase antibody; Tg-AB, thyroglobulin antibody; * significant result.

**Figure A1:**
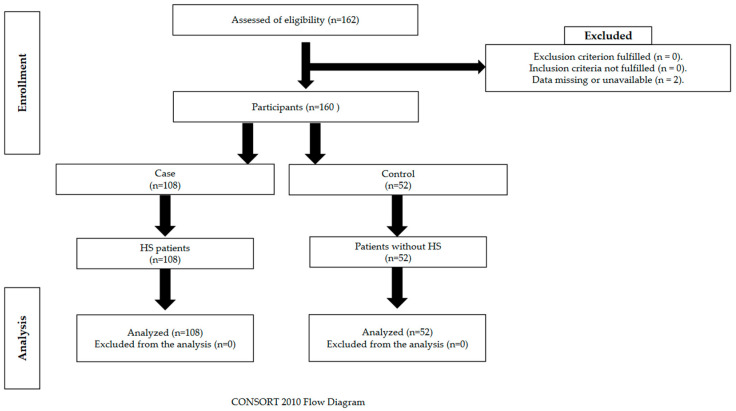
CONSORT flowchart diagram describing the study structure.

## Data Availability

The data presented in this study are available upon request from the corresponding author. The data are not publicly available due to privacy restrictions.
